# Cervicogenic headache: too important to be left un-diagnosed

**DOI:** 10.1186/1129-2377-16-6

**Published:** 2015-01-20

**Authors:** Torbjørn A Fredriksen, Fabio Antonaci, Ottar Sjaastad

**Affiliations:** Department of Neurosurgery, St. Olavs Hospital, Trondheim University Hospital, Trondheim, Norway; Headache Centre, C. Mondino National Institute of Neurology Foundation, IRCCS, Department of Brain and Behavioral Sciences, University of Pavia, Pavia, Italy; Department of Neurology, St. Olavs Hospital, Trondheim University Hospital, NTNU, Trondheim, Norway

**Keywords:** Cervicogenic headache, Headache classification, Unilaterality of headache, Mechanical precipitation of headache

## Abstract

A comparison has been made between the cervicogenic headache criteria in the new IHS classification of headaches (3^rd^ edition- beta version) and The Cervicogenic Headache International Study Group’s (GHISG) criteria from 1998. In a more recent version, the CHISG criteria consist of 7 different items. While “core cases” of cervicogenic headache (CEH) usually fulfill all 7 criteria, the IHS classification - 3^rd^ edition beta version- fulfills only 3 criteria. Although the new three beta version represents an improvement from the previous one, it does not quite seem to live up to the expectations for a diagnostic system for routine, clinical use.

## Status as regards classification of Cervicogenic headache

The present version of cervicogenic headache classification from IHS (3rd edition beta version) [1] is better than the previous one, which mixed- up headache and facial pain. That does not mean that it is flawless. A classification should not only be recognition of disorders, with a minimalist description of some characteristic traits of each disorder. It should also, definitely, serve as a guideline in practical, clinical work, like the IHS criteria do in other disorders in the field, e.g. migraine [1]. That is the main aim of the CHISG criteria [2]. The CHISG and IHS have essentially different approaches to these problems.

## Background

A clinician confronted with a headache patient decides to consult a diagnostic guideline. In the IHS system, he will find that the mode of presentation of criteria seems to differ in primary and secondary headaches. In primary headaches, the headache itself is described in detail, whereas in secondary headaches, like CEH, the (putative) underlying pathology is focused. For example at C I, under “Diagnostic criteria”: “Headache has developed in temporal relation to the onset of the cervical disorder—”. In the actual, clinical situation, this statement will be inutile for all practical purposes. It may concern a situation in the remote future and will accordingly not be very illuminating. One will only rarely be in a position to watch the growth of an underlying pathological process in CEH: such a process presumably develops insidiously slow. C I should probably be removed from the criteria and placed under another heading. C I seems to be some type of writing desk medicine- not a guideline for practical work.

Moreover, in our opinion, headache characteristics (localization, intensity, and duration) should come first, followed by other characteristic traits (precipitation mechanisms, reduction in range of motion etc.) [3].

### Clinical symptoms and signs

The same last as in C I is found in point C 2: “Headache has significantly improved or resolved in parallel with improvement in or resolution of the cervical disorder—”. The usefulness of this criterion is also limited in the diagnostic situation. One exceptional disorder that may seem to fit both C1 and C2 is: “Tractor drivers´ head- and neck-ache” [4]. This headache comes in connection with tractor-driving during chores and fades away after the chore. This headache does not become chronic—the stimulus is turned on and off. ---

Point C3.comes in another category. It, moreover, seems to contain at least *two c*riteria: I. Reduction, cervical range of motion. And II: Significant worsening of headache by mechanical influence. These two criteria are not directly akin. It would, therefore, probably be best to have them under different numbers. As for range of motion, the extent of normalcy should be outlined. Otherwise, this point may not be of optimal value. The magnitude of the stimulus, needed during provocations, should be specified, in order to create a useful criterion. Mechanical precipitation of pain attacks can be obtained in two ways: by external pressure or by positioning the neck in unphysiological positions for a prolonged time. This should probably also have been mentioned under C3.

C3 is important, but in our estimation it needs an upgrading. If left like it is, it will leave the clinician with more questions than advices. ----C4 is unproblematic.

Then, under what is termed “Comments” side-locked pain is mentioned. This is a fundamental quality of CEH. It has not been mentioned previously by IHS. IHS has thus been following our footsteps. Does not bilaterality exist in CEH? It probably does, but then the level of pathology in the neck may be (“is”?) different on the two sides. It is then probably a question of “unilaterality on two sides”. According to the IHS Committee, side-locked pain should not be regarded as “unique” (together with mechanical precipitation procedures and posterior → anterior movement (probably not radiation!) of the pain). All these features are, in our estimation, major criteria of CEH. (Posterior → anterior movement of pain has previously been *sub judice* as a symptom in CEH. It was investigated in 1989 by Fredriksen [5]. However, in the Vågå study, where it was a *free variable*, it proved to be present almost invariably [3, 6]. It has, therefore, more recently been formally recognized as a true CEH criterion (by the CHISG classification committee; TAF & OS: two of the three original members, and by: FA, previous chairman of the group). --- It seems to be a misunderstanding to speak about “unique” criteria. Are the migraine criteria “unique”? Each solitary of them? Of course not. That is not the way headache descriptions are built up. It is the impact of all of them, e.g. pulsating pain, photophobia etc. that viewed together make up the picture of migraine. --Some of the CEH criteria may, nevertheless, be somewhat more specific than the migraine criteria, e.g. unilaterality without sideshift; and pain that starts in the posterior part of the head and then “crawls” to the front.

There exists no gold standard as far as CEH criteria are concerned. Closest to this, probably comes the CHISG diagnostic criteria. These are not even cited in the IHS beta version, so a comparison between them could not be made.

We are highly uncertain as to how the “Comments” should be regarded. Apparently, they are not regarded as criteria, on line with “Diagnostic criteria”. If so, they should primarily have been placed there. This leaves us as readers and future potential users with a considerable dilemma.

The grave question is: does the IHS scheme stand the test? Can CEH diagnosis be made on the basis of this scheme? In connection with the CHISG criteria, we proposed two constellations of diagnostic phenomena, as minimum requirement for the diagnosis.: I: “Confirmatory” combination of criteria for CEH diagnosis and II: “Provisional” criteria, both categories containing 4 solitary, obligatory items; two items overlapped [2]. The IHS criteria do not fulfill any of the two combinations: one criterion is lacking for each of them. If what *we* understand by criteria, in the “Comments” [1] were included, the situation would change.

As already mentioned: In connection with the Vågå study [3, 6], we introduced an enumeration of diagnostic factors, with a total of 7 or 8 factors, depending upon whether diagnostic blockades are incorporated or not:

I Unilateral head pain, without side shift ¤
II Provocation, unphysiological neck positions *
III Provocation, externally; neck/occipital area *
IV Range of motion, neck; deficit *
V Shoulder pain, diffuse
VI Arm pain, diffuse
VII Pain, starting posteriorly- ending up anteriorly ¤
VIII Diagnostic, anesthetic blockades

* IHS diagnostic criteria (C3); n = 3. ¤ “Criteria”, mentioned under” Comments” n = 2; a total of 5 criteria.

Diagnostic blockades are not obligatory in routine work, for which reason they are placed below a line.

## Discussion

The Vågå study showed a close-to-complete congruity between the orthodox application of the criteria [2] and the aforementioned enumeration [3, 6]. This means that the IHS criteria also can be compared with the enumeration criteria. Also in this comparison, the IHS criteria seem to fail: with three out of seven criteria present. If the criteria from the “Comments” section were added, there would be five out of seven CHISG criteria present. In this situation, a CEH diagnosis could have been made. In the first situation, it could definitely not have been made. –The “enumeration” method is easier to apply than the original “orthodox” method. --- In the Vågå study, there were 41 “core” CEH cases; prevalence: 2.2% [6]. The mean number of criteria was close to 6.0; or: 7.0, if the 7th criterion was included. To use only core cases, is probably the best way to calculate CEH prevalence. If cases of co-morbidity with migraine and tension-type headache were added, the prevalence of CEH in Vågå would be: 4.1%. CEH diagnosis is no left hand work, and the diagnostic accuracy is probably reduced in the latter situation. –This, nevertheless, means that in medical practice, one will with not too long intervals encounter CEH patients [7].

## Conclusion

The present IHS criteria version is still probably not a safe basis for diagnosing CEH, although it represents an improvement from the previous IHS version. With the present CHISG criteria, the CEH diagnosis [8] may seem safer than e.g. the migraine diagnosis. It is advocated that the symptoms from the “comments” section of the IHS description are included as criteria to improve the diagnostic accuracy.

### Ethics committee

The Regional committee for medical and health research ethics has approved all our work. After the mid-nineties the Norwegian Data Inspectorate has also approved it.
